# Rapid Dissemination of *Plasmodium falciparum* Drug Resistance Despite Strictly Controlled Antimalarial Use

**DOI:** 10.1371/journal.pone.0000139

**Published:** 2007-01-03

**Authors:** Nitchakarn Noranate, Rémy Durand, Adama Tall, Laurence Marrama, André Spiegel, Cheikh Sokhna, Bruno Pradines, Sandrine Cojean, Micheline Guillotte, Emmanuel Bischoff, Marie-Thérèse Ekala, Christiane Bouchier, Thierry Fandeur, Frédéric Ariey, Jintana Patarapotikul, Jacques Le Bras, Jean François Trape, Christophe Rogier, Odile Mercereau-Puijalon

**Affiliations:** 1 Unité d'Immunologie Moléculaire des Parasites, Centre National de la Recherche Scientifique URA 2581, Institut Pasteur, Paris, France; 2 Hôpital Avicenne, Assistance Publique-Hôpitaux de Paris, Bobigny, France; 3 Unité d'Epidémiologie, Institut Pasteur de Dakar, Dakar, Senegal; 4 Laboratoire de Paludologie/Zoologie Médicale, IRD, Dakar, Senegal; 5 Institut de Médecine Tropicale du Service de Santé des Armées, Marseille, France; 6 Transports Membranaires et Chimiorésistance du Paludisme, Université R. Descartes and Hôpital Bichat Claude Bernard, Assistance Publique-Hôpitaux de Paris, Paris, France; 7 Pasteur Génopole-Ile de France, Plateforme Genomique, Institut Pasteur, Paris, France; 8 Institut Pasteur du Cambodge, Phnom Penh, Cambodia; 9 Faculty of Tropical Medicine, Mahidol University, Bangkok, Thailand; Mahidol University, Thailand

## Abstract

**Background:**

Inadequate treatment practices with antimalarials are considered major contributors to *Plasmodium falciparum* resistance to chloroquine, pyrimethamine and sulfadoxine. The longitudinal survey conducted in Dielmo, a rural Senegalese community, offers a unique frame to explore the impact of strictly controlled and quantified antimalarial use for diagnosed malaria on drug resistance.

**Methodology/Principal Findings:**

We conducted on a yearly basis a retrospective survey over a ten-year period that included two successive treatment policies, namely quinine during 1990–1994, and chloroquine (CQ) and sulfadoxine/pyrimethamine (SP) as first and second line treatments, respectively, during 1995–1999. Molecular beacon-based genotyping, gene sequencing and microsatellite analysis showed a low prevalence of *Pfcrt* and *Pfdhfr-ts* resistance alleles of Southeast Asian origin by the end of 1994 and their effective dissemination within one year of CQ and SP implementation. The *Pfcrt* resistant allele rose from 9% to 46% prevalence during the first year of CQ reintroduction, i.e., after a mean of 1.66 CQ treatment courses/person/year. The *Pfdhfr-ts* triple mutant rose from 0% to 20% by end 1996, after a mean of 0.35 SP treatment courses/person in a 16-month period. Both resistance alleles were observed at a younger age than all other alleles. Their spreading was associated with enhanced *in vitro* resistance and rapidly translated in an increased incidence of clinical malaria episodes during the early post-treatment period.

**Conclusion/Significance:**

In such a highly endemic setting, selection of drug-resistant parasites took a single year after drug implementation, resulting in a rapid progression of the incidence of clinical malaria during the early post-treatment period. Controlled antimalarial use at the community level did not prevent dissemination of resistance haplotypes. This data pleads against reintroduction of CQ in places where resistant allele frequency has dropped to a very low level after CQ use has been discontinued, unless drastic measures are put in place to prevent selection and spreading of mutants during the post-treatment period.

## Introduction

The steady increase of *Plasmodium falciparum* resistance to cheap first line antimalarials over the last decades has resulted in a dramatic increase in malaria-associated morbidity and mortality in sub-Saharan Africa [1], [2]. Research in recent years has established that resistance to chloroquine (CQ), pyrimethamine (P) or sulfadoxine (S) results from the accumulation of multiple mutations in the respective target gene, which once formed, spread across vast, continent-wide areas [3], [4], [5], [6], [7], [8]. The conditions involved in the positive selection of resistant parasites, and the selective pressure contributing to their spread are largely unknown. Malpractice in drug usage is unanimously blamed for permitting emergence of drug resistance, but its impact subsequent spreading of resistance is not known. One reason is the difficulty associated with the assessment of drug intake in endemic areas. Anti-malarial drug pressure is usually inferred from the amount of the drug purchased and distributed in the country, but how this relates to the actual selective forces exerted on the parasite population is unclear, especially since use of antimalarials for any type of fever and often with non optimal drug regimens is widespread [9].

Previous studies have attempted to correlate parasite resistance with antimalarials use at the community level, but even in carefully surveyed settings, irregular compliance and uncertain regimens precluded definitive conclusions [10], [11], [12]. The longitudinal active case detection study launched in Dielmo in 1990, a rural Senegalese village [13], is probably the only place where drug use has been controlled and constantly monitored for more than a decade, coinciding to the time period of expansion of CQ- and SP-resistance across Africa. This is an unprecedented opportunity to quantify the impact of a strictly controlled use of antimalarials on drug resistance. Furthermore, first line treatment was changed in 1995, allowing to explore its consequences on dynamics of spreading of drug resistance.

The design of the Dielmo project involves daily medical surveillance with active case detection, associated with prompt treatment with recommended dosage and duration and monitoring of medication on an individual basis together with the longitudinal recording of transmission [13], [14], [15], [16], [17]. CQ was used in the village as presumptive treatment before the onset of the study, but was replaced by a 3 or 7-day quinine course as first line treatment of all microscopically diagnosed malaria episodes for the first five years of the project [13], [15], [17]. Treatment policy was modified in 1995, with CQ and the sulfadoxine/pyrimethamine combination (SP) being used as first and second line treatments, respectively. Throughout the 1990–9 time period, every treatment course was recorded and clinical efficacy measured by daily monitoring. Parasite isolates were collected on a longitudinal basis. The impact of drug pressure on clinical efficacy, *in vitro* susceptibility and drug target gene flow can thus be accurately quantified.

CQ-resistance *in vivo* has been associated with the presence of a single point mutation at codon 76 (K76T) of the *P. falciparum* chloroquine transporter (*Pfcrt*) [7], [18]. SP-resistance results from point mutations of the target enzyme dihydrofolate reductase (DHFR) and dihydropteroate synthase (DHPS), respectively. Several *Pfdhfr-ts* mutations have been associated with *in vitro* pyrimethamine-resistance, with a key S108N polymorphism that confers in vitro-resistance [19], [20]. Concordant results indicate an association of the triple N51I C59R S108N *Pfdhfr-ts* mutant with reduced therapeutic efficacy [5], [21]. Similarly, several *Pfdhps* mutations have been associated with decreased susceptibility to sulfadoxine *in vitro*. SP therapeutic failures have been associated with presence of the double A437G, K540E mutant associated with the *Pfdhfr-ts* triple mutant [22], [23].

We have analysed here the *Pfcrt, Pfdhfr-ts* and *Pfdhps* loci in a panel of clinical isolates collected every year from 1990 to 1999. We show here that switching to CQ and SP use in 1995 was followed within a few months by a sharp increase in the prevalence of resistance haplotypes, pointing to a remarkably rapid expansion of mutant haplotypes under strictly controlled drug usage. This was associated with a concomitant increase of *in vitro* resistance and with a progressively increased risk of early subsequent clinical malaria episode after a CQ treatment course.

## Materials and Methods

### Study site and design of the survey

Dielmo, located in Sine Saloum, Senegal, is a village of approximately 300 inhabitants, where malaria is holoendemic. Malaria transmission in Dielmo occurs all over the year, unlike the neighbouring villages where it is highly seasonal. In 1990, the entire village population was enrolled in a longitudinal prospective study described in detail elsewhere [13]. Among the population (female: male ratio = 0.98), 20.4% were children less than five years old and 26.8% were 5–14 year-old children. Individual informed consent was obtained from each adult participant and from the parents or legal guardians of all children at the beginning of the study and was renewed on a yearly basis. Any individual could withdraw from the study at any time. Each year the project was reviewed and approved by the Joint Ministry of Health and Pasteur Institute Surveillance Committee [13], [15], [17].

From the end of May 1990 onwards, clinical malaria episodes were diagnosed on site by microscopic examination of blood smears collected for each febrile episode, and prompt anti-malarial treatment administered by a medical team present permanently in the village, who subsequently monitored therapeutic and parasitological efficacy [13], [14], [15], [17].The population of Dielmo was asked not to use any drug without informing the medical team. Random urine tests were made on a regular basis to detect the presence of antimalarials [15]. This indicated the remarkable compliance of the villagers with the study design and anti-malarial intake. For each malarial episode, a fingerpick or 2 mL venous blood sample was collected and stored as a frozen red blood cell pellet at −80°C.

The study period explored here was 29 May 1990–31 Dec 1999. The intensity of malaria transmission was determined by weekly or monthly mosquito collections as described [13]. During this period, the entomological inoculation rate fluctuated from 115 to 347 infective bites/person/year, depending on the year.

### Anti-malarial use

Before the longitudinal survey, CQ was irregularly used as the presumptive treatment, together with traditional medicines. Measures of CQ levels in children were made before the onset of the project using the Bergquist test. This showed that about 13% of the children had detectable CQ levels, similar to children from five neighbouring villages. From 29 May 1990 to 17 Jan 1995, quinine (Quinimax®) administered every 8hr by a medical field worker or a physician for 3 or 7 days was used for 96.4% of the 1722 anti-malarial treatments administered, including a total of 277 eradication therapies in 1992 and 1994 [24], [25]. Halofantrine was used for eradication therapy in 46 adults in 1994 and CQ for three curative treatments, representing 3.42% and 0.17% of anti-malarial treatments administered, respectively. Early in 1995, the treatment policy was changed to CQ (Nivaquine®), and SP (Fansidar®) as first and second line treatments, respectively [17]. CQ was introduced on 20 Jan 1995, and SP was used for the first time on 12 Sep 1995. From 01 Jan 1995 to 31 Dec 1999 quinine, CQ and SP were used for 5.2%, 85.3% and 9.5% of the 3130 anti-malarial treatments administered, respectively. There was no chemoprophylaxis among villagers. The use of antimalarials was restricted to acute malaria attacks in accordance with the following criteria: 1) fever with a parasite:leukocyte ratio ≤2 in children less than 10 years of age, 2) fever with a parasite:leukocyte ratio ≤0.5 in pregnant women, or 3) fever with a positive thick blood film in individuals with symptoms compatible with severe malaria or in individuals returning from an area of low endemicity where they had lived for more than one year during the past three years. When fever persisted the next day, another thick blood film was made. Criteria for antimalarials treatment remained unchanged. However, in the absence of any clinical improvement associated with a parasite density close to the treatment threshold in children and adults, or a positive thick blood smear in pregnant women, the prescription of a second antimalarial treatment was based on the patient's clinical and epidemiological data.

**Figure 1:**
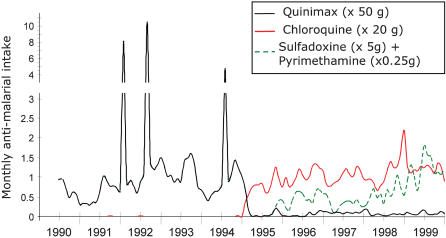
Amount of antimalarials used in Dielmo from 1990 to 1999. The mean number of person-years surveyed was 124 in 1990 (from June to December) and 295, 235, 238, 262.8, 260.1, 257.1, 292.5, 288.7, 299.3 and 297.8 from 1991 to 1999, respectively. The cumulated survey days were 51745, 81120, 82583, 90761, 89551, 88908, 100706, 95917, 100942 and 99083 from 1990 to 1999, respectively. The monthly quinine, CQ and SP intake was calculated from the recorded drug prescriptions in the data base. For <5% of cases, the actual prescribed dose was not available and was extrapolated from the standard treatment course at that time in the village. The peaks in 1992 and 1994 correspond to eradication therapies administered to 100, 118, and 59 persons, respectively [21], [22]. There were 155, 262, 567, 310, 428, 435, 710, 649, 681 and 823 treatment courses administered from 1990 to 1999, respectively.

The yearly intake of antimalarials, which represents drug pressure on the local parasite population, is depicted in Figure 1. We used each calendar year, except for the year 1990 which included the period 29 May–31^st^ Dec 1990. The lack of non-prescribed CQ use by villagers in 1990–94 was ascertained by consistently negative random monthly urine tests [13], [15], [17]. The number of CQ and SP treatments per person per year was calculated based on the number of days of follow up divided by the number of persons enrolled that year. These were as follows : 51,745/253, 81,120/235, 82,583/238, 90,761/263, 89,551/260, 88,908/257, 100,706/293; 95,917/289, 100,942/299, 99,083/298 for 1990 (7 months of follow up), 1991, 1992, 1993, 1994, 1995, 1996, 1997, 1998, 1999, respectively.

### *In vivo* efficacy

The risk of early occurrence of a subsequent clinical malaria episode after a CQ or SP treatment course was estimated by the proportion of cases present in the village for ≥31 days after the treatment in whom the treatment was followed within <7, 14, 21 or 28 days by a second *P. falciparum* high density episode or the need to administer an additional antimalarial treatment. From 1.1 to 7.7% of the treatments were administered for *P. ovale* clinical attacks. None necessitated a second treatment course. They were counted in the overall number of antimalarial treatments administered, but not in the calculation of risk of subsequent clinical malaria episodes that was restricted to *P. falciparum* attacks.

Overall, there were 1899 first CQ treatment courses for which the clinical follow-up was complete for the next 7, 14, 21 and 28 days. There were 127, 316, 587 and 812 second treatments administered within <7, 14, 21 and 28 days, respectively. The 199 SP treatment courses administered all had a complete follow-up for >31 days. There were 5, 6, 16 and 48 cases of administration of another treatment within 7, 14, 21 and 28 days, respectively. The protocol for the longitudinal follow up was not designed as a drug clinical trial, and parasite clearance was not systematically recorded as recommended for trials. The detailed analysis of therapeutic efficacy over this decade using additional criteria (including clearance rates when available and incorporating individual host risk factors, age patterns as well as fluctuations of transmission) is in progress and will be reported elsewhere (Tall et al, in preparation).

### Parasite population surveyed

Since the parasites exposed to heavy drug pressure were those associated with clinical episodes, we studied parasites collected during clinical malaria attacks. The isolates were selected from the existing collection of frozen blood samples as follows: we interrogated the database to identify the samples collected before treatment administration from patients diagnosed with a clinical malaria episode (fever+high parasite density) over the 10 y-period. From a list of approx 3400, 336 samples were chosen for molecular analysis so as to survey the largest possible panel of villagers, but ignoring therapeutic efficacy of the treatment administered after sample collection, and selecting wherever possible isolates with interpretable *in vitro* data, whatever the result of the assay. Since in this holoendemic setting, the heaviest clinical malaria burden is in the <10 y olds and since furthermore some children are more susceptible than others [16], we needed to avoid iteration biases due to increased susceptibility of some individuals. We therefore set a ≥three y interval between two samples from the same individual and set the additional restriction that no person could contribute with more than three samples overall. This reduced the risk of over-representing certain genotypes to which some individuals might be more susceptible than others, and also of over-estimating polymorphism because successive clinical malaria attacks experienced by one person are caused by “novel” parasites [26].

The number of isolates studied each year is shown in Table 1. The samples originated from 246 villagers, with 167, 68 and 11 villagers contributing once, twice and three times, respectively, to the panel of isolates for the retrospective molecular survey. There were 130 males and 116 females among the villagers recruited [175 and 161 males and females, respectively, among the panel of isolates studied]. The mean age at the time of blood sampling was 11.4±13.1 years. The isolates were from 31 out of 34 village compounds.

**Table 1:**
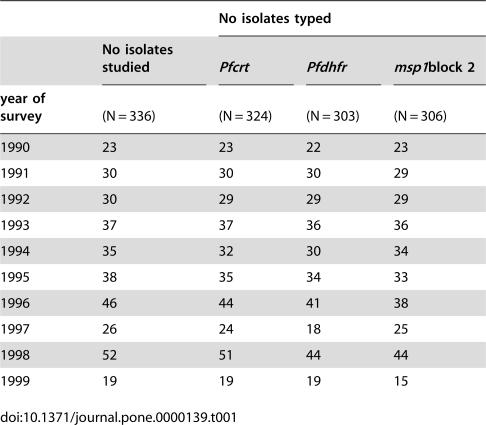
Number of isolates studied by calendar year of survey and successfully typed by molecular beacons for the *Pfcrt* K76T and *Pfdhfr-ts* S108N loci and by nested PCR for the *Pfmsp1* block2 locus.

		No isolates typed		
	No isolates studied	*Pfcrt*	*Pfdhfr*	*msp1*block 2
year of survey	**(N = 336)**	**(N = 324)**	**(N = 303)**	**(N = 306)**
1990	23	23	22	23
1991	30	30	30	29
1992	30	29	29	29
1993	37	37	36	36
1994	35	32	30	34
1995	38	35	34	33
1996	46	44	41	38
1997	26	24	18	25
1998	52	51	44	44
1999	19	19	19	15

### *In vitro* susceptibility assays

Venous blood samples were collected during the high transmission season on a longitudinal basis from consenting patients with clinical malaria. Assays were conducted using the isotopic microtest as described [27], [28], [29] on clinical malaria isolates collected in the village or in the health centre of Toubacouta. The isolates with an IC50 >100 nM and >2000 nM for CQ and pyrimethamine, respectively, were classified as resistant [27], [28], [29].

### DNA extraction

Frozen blood samples were thawed and extracted with phenol-chloroform as described [30] and stored at −20°C until use.

### Molecular beacon genotyping

Molecular beacons for Pfdhfr108 and Pfcrt76 genotyping were used essentially as described [31], [32]. The PCR primer and molecular beacon sequences are shown in Table S1. The molecular beacons reliably detect ≥10% of the alternative genotype. However, in the samples used here the ratio of allele was not greater than 1/3.

### Gene sequencing

Gene sequencing was carried out for the isolates typed by molecular beacons as containing a single allelic form at the locus. All sequence data with ambiguous positions were rejected. The 72-6/220 *Pfcrt* gene sequence was established as described [33], using the primers listed in Table S1. The entire 1.8 kb *Pfdhfr-ts* coding sequence was amplified by PCR in a 50 µL reaction volume containing 2 µL DNA, 10 mM Tris-HCl pH 9.0 at 25°C, 50 mM KCl, 0.1% Triton X-100, 2.5 mM MgCl_2_, 200 µM each dNTP, 0.6 µM each DhfrPfQs and DhfrPfCoasQ primers (see Table S1), with 2.5 U Taq Polymerase (Promega). After an initial denaturation step at 94°C for 3 min, samples were subjected to 5 cycles of denaturation at 94°C for 30 sec and hybridization/extension at 64°C for 4 min followed by 35 cycles of denaturation at 94°C for 10 sec and hybridization/extension at 66°C for 3 min 15 sec, with a final extension step for 8 min. The amplified PCR products were visualized on an 1.5% agarose gel by ethidium bromide staining. A second PCR was performed on the negative samples in a 50 µL volume as above, using 2 µL of the first reaction and 0.8 µM each of dhfrsm13 and dhfrasm13 primers (see Table S1). The nested PCR conditions were: an initial denaturation step at 94°C for 3 min 94°C followed by 25 cycles of denaturation at 94°C for 30 sec and hybridization/extension at 66°C for 4 min, with a final extension for 10 min at 66°C. Direct sequencing was done with forward and reverse external and internal sequencing primers (see Table S1 for primer sequence), using an ABI Prism sequencer 3100 as described [34].

The complete coding region of *Pfdhps* was amplified and sequenced (ABI 3100 Genetic Analyser, Applied Biosystems, Courtaboeuf, France) as described [23].

### *Pfmsp1* block2 genotyping

The highly polymorphic *Pfmsp*1 block 2 was typed by semi-nested PCR in a 50 µL reaction volume containing 5 µL DNA, 50 mM KCl, 1.5 mM MgCl_2_, 10 mM Tris-HCl pH 9.0, 200 µM dNTP, 5 U Taq Polymerase (Amersham Pharmacia), 1 µM of each primer (see Table S1 for primer sequence). For the first PCR, a first denaturation step at 96°C for 5 min was used, followed by 25 cycles of denaturation at 96°C for 1 min; hybridization at 64°C for 1.30 min, extension at 72°C for 45 sec with a final extension at 72°C for 7 min. The semi-nested PCR was carried out using a forward family specific primer and a reverse conserved primer (see Table S1), under the same conditions except that annealing was done at 68°C. PCR products were analysed on 1.5% Nusieve∶agarose gels (1∶3). The size of the bands was evaluated using the 100 bp DNA ladder (BioRad) as size markers. Alleles were classified in 10 bp bins.

### Microsatellite genotyping

Microsatellites were determined from single genotype infections from which we had obtained reliable unambiguous gene sequence at all positions. All ambiguous results were rejected from the analysis. The *Pfcrt* intron 4 microsatellite was analysed by direct sequencing of the PCR fragment generated using primers located within exons 4 and 5 (see primer sequence in Table S1) essentially as described [33]. The various *Pfcrt* intron 4 microsatellite types were assigned codes described in Table S2.

The *Pfdhfr-ts* flanking microsatellite loci called −0.1 kb, and +0.5 kb, as well as for some isolates the −4.4 kb locus, were studied by semi-nested PCR using specific primers described by Roper et al 2003 [5] and Nair et al, 2003 [35] (primer sequences shown in Table S1) but using different experimental conditions as follows. The primary and secondary mixture reactions for the −0.1 kb and −4.4 kb loci were carried out in a 50 µL reaction volume containing 10 mM Tris-HCl pH 9.0, 50 mM KCl, 0.1%Triton®X-100, 2.5 mM MgCl_2_, 0.64 µM dNTP, 0.05 U Taq polymerase (Promega) and 1 µM each primer. The reactions were cycled as follows: denaturation at 95°C for 5 min, followed by 35 cycles of denaturation at 95°C for 30 sec, annealing at 58°C for 45 sec and extension at 72°C for 30 sec, followed by a final extension at 72°C for 7 min, then held at 4°C. The fluorescent labelled primer was added in the secondary reaction using the same reaction cycles. For the +0.5 kb locus, the primary and secondary PCR were done as above except that MgCl_2_ was used at 1.5 mM and that annealing was done at 48°C for the first reaction and at 53°C for the secondary PCR. For microsatellite genotyping, 1 µL of diluted PCR products was added to 10 µL of a formamide/ROX-labeled internal size standard (Genescan® 400HD [ROX], Applied Biosystems) mixture (82:1), then processed on an ABI Prism 3700 automated DNA sequencer for electrophoresis. Data were imported to GeneScan software to perform fragment analysis by sizing each peak relative to the internal standard. The results were imported to Genotyper software to provide final results in allele calls as well as automated table building. Reference fragments from 3D7 and FCC1 were included as standards in each amplification experiment. The 3D7 and FCC1 *Pfdhfr-ts* haplotypes are described in Table S3.

### Statistical analysis

Allelic frequencies and multiplicity of infection were compared by the exact Fisher and Kruskall-Wallis tests, respectively. We first considered each calendar year separately and subsequently grouped together consecutive years that were not statistically different. The temporal *Pfcrt* genotype distribution could be divided into three main distinct periods, namely 1990–2, 1993–4 and 1995–9. The temporal *Pfdhfr-ts* genotype distribution showed four distinct periods namely1990–92, 1993–4, 1995 and 1996–9.

Mean and median age distribution by *Pfcrt* or *Pfdhfr-ts* genotype were analysed by the Kruskall-Wallis test. Genetic diversity was assessed by the number of alleles per locus and Nei's unbiased expected heterozygosity (He) [36] from haploid data using FSTAT version 2.9.4. (Goudet J. (2003) update from [37]). Differences between the estimated He at each locus were tested by the Wilcoxon signed rank test using STATA 9 software (Stata Corporation, College Station, TX, USA). The selection coefficient (*s*) was calculated as described by Nair et al [35] from the slope obtained by plotting Ln(R/S), with R and S being the frequency of the resistant and sensitive allele, respectively, against the number of estimated parasite generations. For CQ-selection, R and S were calculated from the K76T *Pfcrt* molecular beacon genotype based on prevalence of infected individuals for each allele. *S,* which indicates the relative survival of resistant and sensitive parasites at each generation, was calculated using two estimates for parasite generation, namely 2 or 4 months i.e. 6 to 3 generations per year [38]. The fitness (*f*) of resistant parasites was estimated by *w* = 1+*s*. Linkage disequilibrium was tested using GENEPOP V3.3 [39]. The null hypothesis was the independence of the genotypes at one locus from genotypes at the other locus. GENEPOP creates contingency tables for all pairs of loci in each period, then performs a probability test (or Fisher's exact test) for each table using a Markov chain [40]. The frequencies of early occurrence of a subsequent clinical malaria episodes after an anti-malarial treatment were compared using exact Fisher's tests.

## Results

### Resistance mutation frequency assessed by molecular beacons

**Figure 2:**
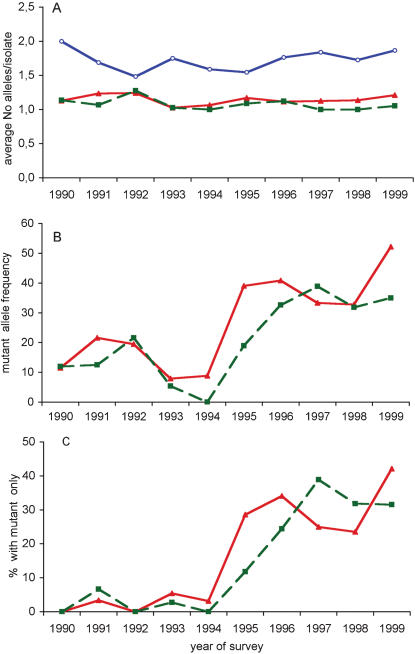
Temporal variation of the multiplicity of infection in Dielmo (A), frequency of Pfcrt codon 76 and *Pfdhfr-ts* codon 108 genotypes (B) and frequency of infections with only mutant type detected (C). The number of isolates typed at each locus is indicated in Table 1. (A). Multiplicity of infection is depicted separately for each locus. For *Pfmsp1* block2, the figures derive from nested PCR analysis using family-specific primers and allele identification based on allelic family assignment and size polymorphism. For *Pfcrt* and *Pfdfhr-ts*, it is based on K76T and S108N genotypes determined by molecular beacons, respectively. Symbols used: (Red triangles): *Pfcrt* codon 76 genotype; (green squares): *Pfdhr-ts* codon 108 genotype, (blue open circles) *Pfmsp1* block2. B) Allelic frequency of resistance genotypes, calculated as percentage of mutant genotype within the total number of alleles detected for each locus. Symbols used as in A. C) Percentage of isolates containing only the mutant type. Symbols used as in A.

The *Pfcrt* K76T and *Pfdhfr-ts* S108N genotypes, which are key to resistance to CQ and pyrimethamine, respectively, were established for 324 isolates (151 from 1990–1994 and 173 from 1995–1999) and 303 isolates (147 from 1990–1994 and 156 from 1995–1999), respectively. The K76 and 76T *Pfcrt* genotypes were detected in 269 and 100 isolates, respectively. The wild type S108 and mutant 108N *Pfdhfr-ts* were detected in 259 and 67 isolates, respectively. Multiple infections, i.e. infections where both mutant and wild type codons were present, accounted overall for 14% (45 of 324) and 7.6% (23 of 303) of the isolates for *Pfcrt* and *Pfdhfr-ts,* respectively. The average number of alleles per isolate did not vary for the *Pfcrt* locus over the 10y period (Kruskall-Wallis test, p = 0.18) (Figure 2A). In contrast, it showed significant temporal variations for the *Pfdhfr-ts* locus (Kruskall-Wallis test p = 0.001, Fisher's exact test, p = 0.001) (Figure 2A).

The *Pfcrt* allele distribution varied significantly over time. The proportion of isolates harbouring a mutant 76T (either single or associated with the K76 allele) decreased from approx. 22% in 1990–92 to 8.7% in 1993–94, increased to 44% in 1995 and subsequently remained at a similarly elevated level (Fisher's exact test, p = 0.004 and <0.001, respectively) (Figure 2B). Thus, the change for CQ in 1995 was followed by a marked increase in the prevalence of the mutant allele. It was also associated with an augmented prevalence of isolates containing the 76T mutant type only, which raised from 4.3% in 1993–94 to 29.5% in 1995–99 (Fisher's exact test, p<0.001; Figure 2C). During the year 1995, the selection coefficient of the 76T mutants was 0.22 and 0.44, assuming 3 and 6 generations/year, respectively.

The frequency of mutant 108N *Pfdhfr-ts* genotypes (either single or in multiple infections) decreased from 18.5% in 1990–92 to 3% in 1993–94 (Fisher's exact test, p = 0.021), increased to 20% in 1995 and again to around 35% in 1996–99 (Fisher's exact test, p<0.001) (Figure 2B). This increase was associated with an increasing proportion of isolates typed as containing only the 108N mutant that increased from 1.5% in 1993–94 to 11.8% in 1995 (Fisher's exact test, p = 0.043) and further increased to 30.3% in 1996–99 (Fisher's exact test, p = 0.030) (Figure 2C). Thus, in 1990–94, the 108N mutation was detected mainly in mixed infections, whereas it was present mostly in isolates containing that single allele in 1995–99.

### Multiplicity of infection

To test whether drug policy has impacted on the overall multiplicity of infection, we genotyped *Pfmsp1* block 2, a highly polymorphic locus derived from a merozoite surface antigen, unrelated to the CQ or SP target genes and frequently used for assessing multiplicity of infection [26], [30]. *Pfmsp1* block2 genotypes were established for 306 isolates. This highlighted 38–66% multiple infections, depending on the year. The mean number of distinct *Pfmsp1* block2 alleles detected for each isolate did not show significant fluctuation over the years (Kruskall-Wallis test, p = 0.51) (Figure 2A). This indicated that the various drug regimens implemented in the village throughout the 10y period did not profoundly affect the average number of parasite clonal types associated with clinical malaria.

### Genotyping by direct sequencing

To look for the presence of additional mutations in the target genes and establish haplotypes, we sequenced PCR-amplified gene fragments or full length genes. For *Pfcrt*, we analysed the gene region coding for the resistance haplotype, where the 76T mutation is associated with additional 74I 75E mutations also encoded by exon 2, together with a 220S mutation encoded by exon 4 [7]. The 74-76/220 haplotype was determined for 21 isolates with a 76T mutation by amplification and sequencing. All isolates harboured the CVIET/S haplotype. No novel mutation was observed in this region (data not shown).

**Figure 3:**
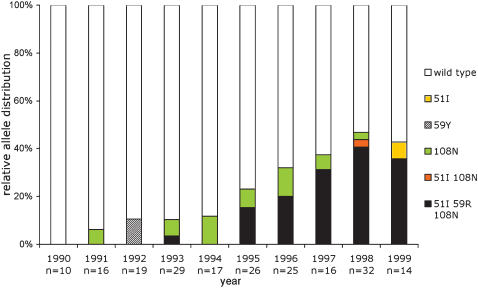
Temporal variation of the relative *Pfdhfr-ts* gene polymorphism in Dielmo during 1990–99. The 1.8 kb PCR fragment corresponding to the full length *Pfdhfr-ts* coding sequence was sequenced on both strands for a total of 204 isolates. The yearly distribution of the various genotypes is shown, using the colour code shown to the right of the figure. The alleles presenting synonymous mutations were omitted from the colour coding. The C59Y non-synonymous substitution was a TGT to TAT mutation. The synonymous mutations (CTA to TTA or CTC for codon 40, GGA to GGC for codon 241) are not depicted. No mutation was detected at codon 16 and 164, and no *bolivia* repeat type was observed. Overall there were 155 isolates with wild type coding sequence and 49 isolates with non synonymous mutations (76% and 24%, respectively). There were 15 (7.3%) single mutant*s* [51I (0.5%), 59Y (1%), 108N (5.8%)], one (0.5%) 51I 108N double mutant and 33 (16.2%) 51I 59R 108N triple mutants.

A 1827 bp *Pfdhfr-ts* full gene sequence was generated for 204 isolates (91 from 1990–1994 and 113 from 1995–1999). This identified three previously reported single nucleotide polymorphisms [SNP] (51I, 59R and 108N) and four novel SNPs within the *Pfdhfr-ts* coding region [59Y, and two synonymous L40L and one G241G]. The *ts* coding region was identical in all samples. Overall there were nine distinct alleles at the nucleotide level, including four wild type alleles, three of which with synonymous substitutions. Hence at the protein level, there were six distinct alleles: wild type, single mutant (51I or 59Y or 108N), double mutant (51I 108N) and triple mutant (51I 59R 108N) alleles. It is noteworthy that the 59R SNP was not detected as a single or double mutant, but was observed only in the triple mutant haplotype. Their temporal distribution is shown in Figure 3. There was no significant increase of any single mutant after SP introduction in the village in 1995. However, the 51I 59R 108N triple mutant frequency increased from 1.1% in 1990–94 to 15.4% in 1995 and further increased to 32.2% in 1996–1999 (Fisher's exact test, *p*<0.001).

Analysis of *Pfdhps* by sequencing was done for 27 isolates collected in 1997–1999. This identified one S436F (3.7%), one S436A (3.7%) and 9 A437G (33%) mutants. The other positions, including codon 540 usually associated with SP-resistance [22] were wild type. All alleles sequenced were single mutants (data not shown). So, overall there were four distinct *Pfdhps* alleles detected in this analysis.

### Analysis of genetic relatedness of resistance mutants using microsatellite genotyping

**Figure 4:**
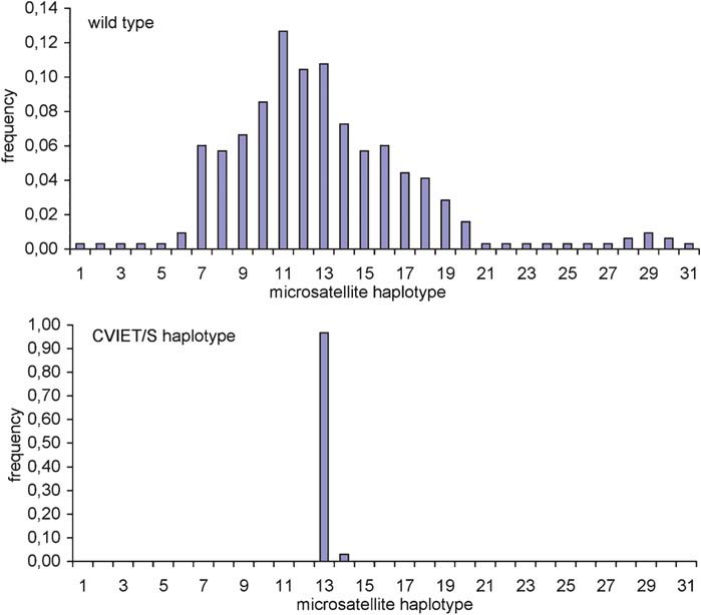
Frequency distribution of *Pfcrt* intron 4 microsatellite types by codons 72–76 and 220 haplotype. 247 isolates were typed (19, 23, 22, 33, 28, 22, 29, 17, 39 and 15 isolates in 1990, 1991, 1992, 1993, 1994, 1995, 1996, 1997, 1998 and 1999, respectively) for the intron 4 microsatellite by gene sequencing (see Materials and Methods). There were 31 CVIETS haplotypes and 216 wild type haplotypes. The haplotype codes are listed in Table S2.

To analyse the genetic relatedness of the alleles carrying the CVIET/S haplotype, we sequenced the exon4-exon5 *Pfcrt* gene region encompassing the intron 4 microsatellite, located just downstream from codon 220 [3], [7]. This was done for 247 isolates. No novel mutation was detected in the exons. Thirty one microsatellite types were observed. Their distribution was highly skewed (Skewness-kurtosis tests for normality, *p*<0.001) (Figure 4). All but one CVIET/S alleles were associated with a type 13 microsatellite [(TAAA)_3_(TA)_15_], i.e. presented the characteristic intragenic signature of the resistance allele of Southeast Asian origin [33]. One CVIET/S allele was associated with the related type 14 microsatellite [(TAAA)_3_(TA)_16_] (unbiased expected heterozygosity He = 0.07, n = 31). In contrast, the CVMNK/A wild type coding sequence was associated with 31 distinct microsatellite types (Table S2), depending on the isolate (unbiased expected heterozygosity He = 0.93, n = 318).

**Figure 5:**
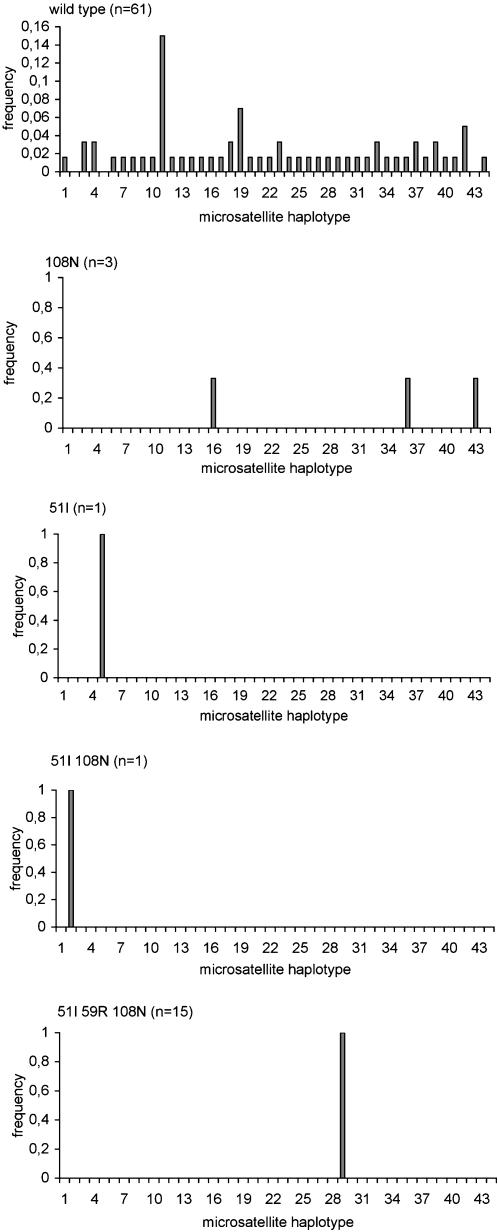
Frequency distribution of *Pfdhfr-ts* −4.4 kb/−01 kb+0.5 kbmicrosatellite haplotypes by coding the sequence mutant type. The microsatellites were determined as described in Materials and Methods for 81 isolates. The haplotype codes are listed in Table S3.

To study *Pfdhfr-ts* gene flow within the village, flanking microsatellites were analysed. We typed 81 isolates for the −0.1 kb and +0.5 kb microsatellite loci, including 38 for the additional −4.4 kb microsatellite locus (Figure 5 and Table S3). This showed that the *Pfdhfr-ts* wild type coding sequence was associated with 41 distinct microsatellite combinations. Each microsatellite locus was highly polymorphic [unbiased expected heterozygosity He = 0.88, 0.89 and 1.00, n = 73, 62 and 5 for the −0.1, +0.5 and −4.4 kb loci, respectively. Heterozygosity across the three loci (mean±SD) = 0.88±0.01]). There were a minimum of three distinct haplotypes for the 108N single mutants [for three 108N mutants, the +0.5 kb microsatellite could not be determined, but each had a different −0.1 kb/−4.4 kb microsatellite haplotype] and each of the single 51I and 51I 108N double mutants was associated with a distinct microsatellite combination [heterozygosity across the three loci for the single or double mutants (mean±SD) = 0.82±0.1]). In contrast, all 51I 59R 108N triple mutants were associated with a single microsatellite allele at each locus [He = 0.00, 0.00 and 0.00, N = 16, 15 and 26 for the −0.1, +0.5 and −4.4 kb, respectively, heterozygosity across the three loci: (mean±SD) = 0.00±0.00]. Thus, there was a single tri-microsatellite locus haplotype associated with the *Pfdhfr-ts* triple mutant. This resistance haplotype is most likely the same as the resistance allele of Southeast Asian origin described in KwaZulu Natal and Tanzania in 1995–9 [5], [6], since they have microsatellite profiles that can be regarded as identical, considering inter-laboratory experimental differences inherent to microsatellite typing.

There was no significant linkage disequilibrium between *Pfcrt* and the *Pfdhfr-ts* coding sequence and/or flanking microsatellites (Fisher's exact test, each p-value >0.00119, i.e. the adjusted p-value for 5% nominal level). As predicted and confirming the above analysis, *Pfdhfr-ts* and its flanking microsatellites were in strong linkage disequilibrium (Fisher's exact test, p = 0.00024 for each pair of loci)

### Allele distribution by age

While there was no influence of blood group or gender on *Pfcrt* and *Pfdhfr-ts* allele distribution, there was an influence of age. Table 2 shows that isolates with only the *Pfcrt* 76T mutation detected were from younger individuals than those with the wild type K76 allele or those containing both K76+76T mutations (Kruskall-Wallis test; Chi-squared = 6.28 with 2 d.f., p = 0.043). The *Pfdhfr-ts* allele distribution was also age-associated, with the triple mutant detected at a younger age than the wild type, single and double mutants (Kruskall-Wallis test; Chi-squared = 6.7 with 2 d.f., p = 0.035). The S108N genotype distribution showed no association with age (p = 0.283).

**Table 2:**
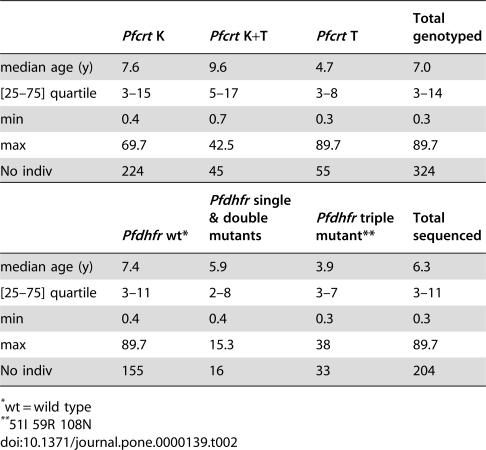
Age association of mutant *Pfcrt* and *Pfdhfr-ts* prevalence over the entire survey period.

	*Pfcrt* K	*Pfcrt* K+T	*Pfcrt* T	Total genotyped
median age (y)	7.6	9.6	4.7	7.0
[25–75] quartile	3–15	5–17	3–8	3–14
min	0.4	0.7	0.3	0.3
max	69.7	42.5	89.7	89.7
No indiv	224	45	55	324

	*Pfdhfr* wt*	*Pfdhfr* single & double mutants	*Pfdhfr* triple mutant**	Total sequenced
median age (y)	7.4	5.9	3.9	6.3
[25–75] quartile	3–11	2–8	3–7	3–11
min	0.4	0.4	0.3	0.3
max	89.7	15.3	38	89.7
No indiv	155	16	33	204

* wt = wild type

** 51I 59R 108N

### Drug pressure and selection of resistance

**Figure 6:**
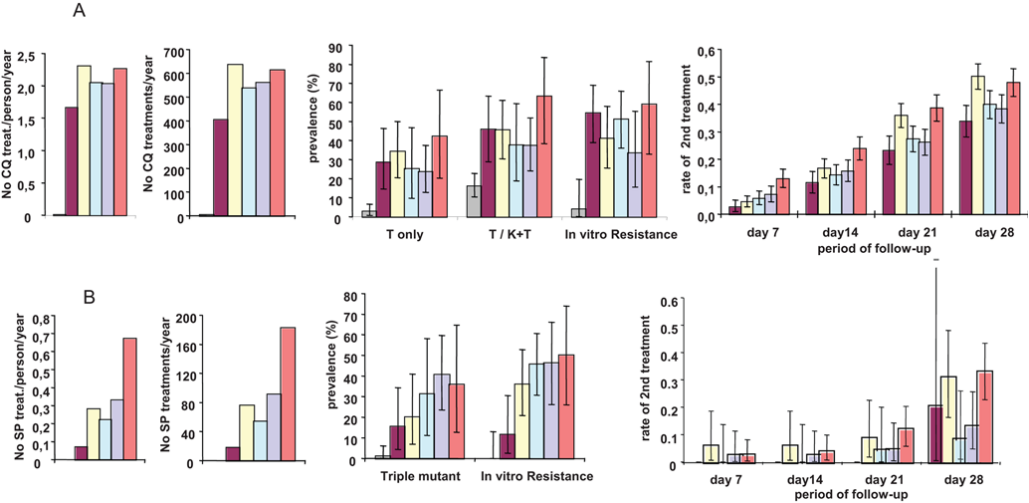
Temporal distribution of CQ and SP drug pressure and drug resistance in Dielmo in 1990–9. The drug pressure is expressed as No of treatments/person/year (first graph) and as overall No of treatment courses administered per year (second graph). Panels A and B refer to CQ and SP, respectively. The prevalence of the *Pfcrt* mutant alleles was calculated from molecular beacon studies (N = 324) (see Figure 2), while the prevalence of the *Pfdhfr-ts* triple mutant was calculated from the full gene sequences available (N = 202) (see Figure 3). *In vitro* susceptibility assays were carried out in 1990–4 during the rainy season (N = 26) and from 1995 onwards for the last 2–3 months of the year, namely from 7/11/1995–26/12/1995 (N = 46) ; 6/01/1996–3/12/1996 (N = 59); 27/10/97–15/121997 (N = 26) ; 10/01/1998–15/11/1998 (N = 54) and 29/09/1999–08/11/1999 (N = 25). The proportion of interpretable CQ and pyrimethamine susceptibility tests was 68–81% and 72–81%, respectively, depending on the year. The prevalence of resistance is expressed as the percent of interpretable assays presenting a CI_50_ for CQ >100 nM or a CI_50_ for pyrimethamine>2000 nM. The occurrence of a second clinical malaria episode within 7, 14, 21 and 28 days of treatment was calculated as described in Materials and Methods. The bars correspond to the 95% confidence interval. The years before implementation of CQ and SP (1990–4) are grouped together. A. CQ pressure, *Pfcrt* 76T resistance mutation, CQ *in vitro* resistance and prevalence of clinical attacks following a CQ treatment B. SP pressure, *Pfdhfr-ts* triple mutant, pyrimethamine *in vitro* resistance and prevalence of clinical attacks following a SP treatment Colour codes: 1990–4: grey; 1995: purple; 1996: yellow; 1997: light green; 1998: light blue; 1999: orange.

Figure 6 A shows the temporal increase of resistance vis-à-vis CQ pressure in the village. During year 1995, 404 CQ treatment courses were administered, corresponding to 1.66 treatment/person/year. During the following years, there was an average of 2.16 CQ courses/person/year and overall 2,345 CQ treatment courses in 1996–9. *In vitro* susceptibility was assessed sporadically over 1990–4 and systematically over a few month period at the end of each calendar year over 1995–9. *In vitro* resistance to CQ, which was low (3.8%) over 1990–4 was markedly increased by the end of 1995, and fluctuated around 45–50% thereafter. The temporal variation of *in vitro* resistance to CQ paralleled the prevalence of *Pfcrt* “76T only” infections. The clinical impact of treatment was estimated by the necessity or not to administer a second treatment course within the <7, 14, 21 and 28 days after the end of the treatment. Occurrence of clinical malaria episodes within 7 days of CQ treatment progressively increased with increasing cumulative number of CQ treatments in the village, resulting in a 5 fold increase in 1999 compared to 1995 (Fisher's Exact test, p<0.001). Such a regular increase was not observed for the longer follow-up periods, nevertheless the incidence of second episodes within 14 days of CQ treatment was doubled in 1999 compared to 1995 (Fisher's Exact test, p<0.001), and it was increased by 1.67- and 1.41-fold by 21 and 28 days, respectively (Fisher's Exact test p<0.001 each). The prevalence of episodes within 14 days and more markedly within 21 and 28 days paralleled the prevalence of the isolates with only the Pfcrt 76T mutant (Figure 6A).

The temporal increase of resistance vis-à-vis SP pressure is shown in Figure 6B. The number of SP treatments/person/year increased from 0.07 in 1995 to 0.5 in 1998–9, corresponding overall to 424 SP treatments administered in the village during 1995–9, and a mean of 0.31 treatments/person/year. *In vitro* resistance to pyrimethamine progressively raised from 14% in 1995 to 50% in 1998 and 1999 with increasing pressure (Figure 6B). There was a strong correlation between the increase in *Pfdhfr-ts* triple mutant frequency and the increasing SP drug pressure during that period (R^2^ = 0.9, with a linear model). Overall, 75 of 199 SP treatment courses were followed by a second treatment (5, 6, 16 and 48 within <7, 14, 21 and 28 days, respectively). Confidence intervals were wide, due to the small number of events each year. The occurrence of 2nd treatment within 21 and 28 days fluctuated between 1996 and 1999 and no temporal trend could be conclusively established.

### Comparison with neighbouring villages

To compare the situation in Dielmo with the surroundings by end of 1995, CQ-*in vitro* tests were conducted during the period Oct 1995–mid Jan 1996 in the health centre of Toubacouta, which captures 11 neighbouring villages with typical CQ-usage. This showed different proportion (Chi2, p<0.005) and level of resistance (log transformed ANOVA p<0.05) between the two sites. In Dielmo, there was approx 60% CQ-resistance (N = 43 samples; mean IC_50_ for CQ = 102 nM; 95% CI = 72.8–143.4 nM; min = 25 nM, max = 563 nM), compared to 24% in Toubacouta (N = 50 samples, mean IC_50_ for CQ = 53.6, 95% CI = 43.2–66.4 nM, min = 25 nM, max = 261 nM).

## Discussion

Here, we have explored whether a strictly controlled use of CQ and SP, administered for microscopically diagnosed, high density malaria episodes would prevent spreading of resistance. Our data indicate that such a drug policy failed to do so. The reintroduction of CQ after several years of discontinued use was followed by rapid dissemination of the *Pfcrt* resistance allele of Southeast Asian origin and of CQ-resistance *in vitro* and progressive deterioration of its clinical efficacy. Within four years of SP introduction in the village as second line drug, *in vitro* pyrimethamine-resistance had markedly raised, *Pfdhfr-ts* resistance genotypes had reached the same prevalence as CQ-resistance genotypes and a similar trend was observed for the A437G *Pfdhps* mutant.

It took 407 CQ treatments in the community (1.66 treatment/person/year) to raise the *Pfcrt* 76T mutation from a 8–9% prevalence in 1993–4 to 46% during the year 1995. Increased pressure (2752 CQ treatments cumulated during the years 1995–9) did not increase the prevalence of the mutant much further, did not increase the *in vitro* resistance further either, but increased by five-fold the rate of treatment failures within 7 days. It took 112 SP treatments in the community (Sep–Dec 1995–year 1996) to raise the prevalence of the triple *Pfdhfr-ts* mutant from 0% in 1994 to 20% in 1996. The pressure on the parasite population was altogether strong because of the very high proportion of high density episodes treated through active medical surveillance and essentially restricted to the 0–14 y age group who accounted for 47% of the village population and experienced 93% of the clinical malaria attacks [13], [16]. Thus, an overall remarkably limited number of treatments in a fraction of the population resulted in massive changes over a very short time period.

The clinical impact of the drug policy implemented in the village for ten years was evaluated by measuring the need for an additional treatment during the one month post-treatment period. The incidence of clinical malaria within 7 days of CQ treatment raised from 2.6% in the year 1995 to reach 13% in the year 1999. This represents a marked increase compared to the 1% incidence by day 7 post-Quinine treatment in the period 1990–3 [41]. These episodes should therefore be classified as early treatment failures. The incidence of early treatment failure after CQ implementation in 1995 increased rapidly, but much slower than the increase of *in vitro* resistance and increase in 76T mutant frequency. Increase of mutant prevalence and/or of *in vitro* resistance are thus relevant indicators of emerging therapeutic failures.

Since CQ half-life is 10–30 days, the parasites that reached high density during the 14–28 day post-treatment period had to survive sub-therapeutic post-treatment drug levels. The increased incidence of a second episode within 14, 21 and 28 days of a CQ treatment from 1995 to 1999 correlating with increasing of the 76T *Pfcrt* mutant frequency is consistent with selection of parasites (be they recrudescence or re-infection) during the post-treatment period. The children were administered several treatments each year [14], [16], and consequently had quite substantial post-treatment time periods, providing the terrain for selecting resistance haplotype among the re-infecting parasites. This is in line with the observed higher frequency of the resistance allele in the younger age group. The dark side of the treatment policy used with properly dosed and administered CQ may thus be to help spreading of reinfecting parasites with resistant genotypes selected during the post treatment period.

The SP pressure was substantially lower than that of CQ and yet similar conclusions can be drawn. There was quite efficient expansion of *in vitro* resistance to pyrimethamine associated with dissemination of the *Pfdhfr-ts* triple mutant, consistent with its reported increased transmissibility [42]. The incidence of early treatment failures was marginal. This is not surprising since SP therapeutic failures have been associated with presence of the “quintuple mutant”, namely the *Pfdhfr-ts* triple mutant associated with the *Pfdhps* A437G K540E double mutant [22], [23], which was not detected in the village. Similar observations were reported for isolates collected in different localities across Senegal in the years 2000–3 [43], [44] This confirms the time shifted invasion by *Pfdhfr*-*ts* and *Pfdhps* alleles observed in East Africa, with invasion of the triple mutant *Pfdhfr-ts* preceding invasion by the *Pfdhps* double mutant that is associated with SP-treatment failure [5], [23]. The use of antibiotics such as Sulfamethoxazole+Trimethoprim (Bactrim™), which may contribute to antifolate resistance [45], [46], [47] was restricted in Dielmo, and furthermore did not substantially vary over the 1990–9 period. This indicates a key role for SP pressure in antifolate resistance in the village. The only mutant *Pfdhfr-ts* genotype that expanded under SP drug pressure had the typical molecular signature of the ancestral resistance haplotype of Southeast Asian origin that has spread across Africa [5], [6]. Several *Pfdhfr-ts* point mutations were detected, including novel ones not observed in other Senegalese sites [43], [44]. None expanded under SP pressure. Each single mutation was associated with a distinct microsatellite haplotype. This is consistent with, but by no means a proof of local generation of mutants. The consequence of the C59Y mutation on enzyme activity is unknown [48].

There was a considerable slowing down of the invasion process after an explosive rapid first phase, despite continued drug pressure. We interpret this as a consequence of having restricted drug pressure to a fraction of the Dielmo population and hence of the parasite reservoir. Thus, older children and adults perpetuated a large parasite population that remained essentially without drug pressure. It is most likely the combination of efficiently treating only a fraction of the potential parasite transmitters with the strong overall population immunity that has slowed down further spreading of resistant mutants in this setting.

Where do the resistant parasites that disseminated after CQ and SP implementation come from? Most probably from local expansion of the residual low frequency resistance alleles and from invasion of resistant strains from the surroundings. Transmission is perennial in Dielmo due to the presence of permanent mosquito breeding sites [13], unlike the surroundings, where it is highly seasonal [49]. Based on the entomological inoculation rate, the villagers' habits during the mosquito biting time periods and the geographic distance from breeding sites outside the village, we estimate that 97% of the infections in adults and 99% of the infections in young children are contracted within the village. The surrounding area was certainly infested with CQ-resistance by 1995, as indicated by the *in vitro* resistance data from the health centre of Toubacouta. The relative contribution of expansion of residual alleles from within the village and invasion of alleles from the neighbourhood during the rainy season is uncertain. Dissection of the phenomenon requires additional population genetic studies.

The Dielmo unique longitudinal assessment at the community level during 10 years of clinical malaria incidence, malaria treatment, drug use, genetics of parasites, travels of individuals, anopheline density and entomological inoculation rates, provides the first autopsy of the spread of drug resistance in an African setting. This indicates that a population unit cannot deter the rapid spread of CQ- and SP-resistance when resistance alleles are present (even at low rate) within the village or in the vicinity, and this even when drug use is strictly reserved for the treatment of properly diagnosed acute malaria attacks and essentially in children. This argues against deploying antimalarials in areas where resistance haplotypes are present, even at low frequency, unless drastic measures are taken to prevent their spreading. Recent report that CQ is again efficacious against clinical malaria in Malawi after discontinued use for more than 1 years has raised hopes for a future reintroduction of CQ in this country [50], [51]. Reintroducing chloroquine is conceivable only once CQ-resistance has totally disappeared or if its transmission is completely blocked. Neither of these conditions are met. CQ-resistance has invaded the whole African continent, although it tends to decline in countries that have discontinued its use [50], [52], [53]. Using chloroquine in combination therapy requires a companion drug that efficiently prevents selection of reinfecting CQ-resistant parasites during the post-treatment period, otherwise spreading of CQ resistance will turn the drug combination into monotherapy. Artemisinin derivatives reduce gametocyte production [54] but their a very short half life is unlikely to prevent selection of re-infecting CQ-resistant parasites during the post-treatment period. A further important conclusion of our study is that greater attention should be paid in drug development to design molecules that target sexual stages as well, and in control efforts to prevent dissemination of the resistance haplotypes, which once formed, will have their spreading promoted by drug pressure.

## Supporting Information

Table S1 Sequence of the primers and probes used.
(0.07 MB PDF)

Table S2 *Pfcrt* intron 4 Microsatellite haplotypes.
(0.05 MB PDF)

Table S3 Pfdhfr flanking microsatellite haplotypes.
(0.06 MB PDF)
