# Supplementary material for: Rapid Dissemination of Plasmodium falciparum Drug Resistance Despite Strictly Controlled Antimalarial Use
Source: PLoS One. 2007 Jan 3;2(1):e139. doi: 10.1371/journal.pone.0000139 (PMC1764034; doi:10.1371/journal.pone.0000139)
Supplement: Table S1 — Sequence of the primers and probes used. (0.07 MB PDF) [file pone.0000139.s001.pdf]

Supplementary file Table S1.

Sequence of the primers and probes used

Molecular beacons *Pfcrt K76T*	
crtsense (170-190)	5′ TTTAGGTGGAGGTTCTTGTC
crtantisense (368-348)	5′ AATAAAGTTGTGAGTTTCGGA
wild-type beacon K76	5′ GCGACGTGTAATGAATAAAATTTTTGGTCG
mutant beacon K76T	5′ GCGACGTGTAATTGAAACAATTTTGTCGC
Molecular beacons *Pfdhfr-ts S108N*	
dhfrsense (303-326)	5′ TGTGGATAATGTAAATGATATGCC
dhfrantisense (421-398)	5′ CATTTATCCTATTGCTTAAAGGTT
wild-type beacon S108	5′ GCGACGAAGAACAAGCTGGGAAAGGTCGC
mutant beacon S108N	5′ GCGACGAAGAACAAACTGGGAAAGGTCGC
Pfcrt PCR for exon 2 sequencing	
Crt76-5	5′ GGTGGAGGTTCTTGTCTTGG
Crt76-3	5′ ATAAAGTTGTGAGTTTCGGATG
Pfcrt exon 4- exon 5 PCR	
CRT220-sense	5′ TTATACAATTATCTCGGAGCAG
CRT220-antisense	5′ CATGTTTGAAAAGCATACAGGC
Pfdhfr-ts gene sequencing	
DhfrPfQS	5′ CTCGAGGAATTCGGATCCTATGATGGAACAAGTCTGCGAC
DhfrPfCoasQ	5′ TCTAGAAAGCTTGGATCCTAAGCAGCCATATCCATTGAAATTT
dhfrsm13	5' AACAGCTATGACCATGCCATATGTGCATGTTGTAA
dhfrasm13	5' GTAAAACGACGGCCAGTATTAAGCAGCCATATCCATTG
internal primers for sequencing	
DHb-2.b	5′ CTCGAGGAATTCGGATCC
DHg-1.g	5′ TCTAGAAAGCTTGGATCC
Microsatellites	
-0.1kb dhfrts*	
DHFR0.3kb.F	5′ ATTCCAACATTTTCAAGA
DHFR0.3kb.3R	5′ GGCATAAATATCGAAAAC
DHFR0.3kb.RHEX	5′ TCCATCATAAAAAGGAGA
+0.5kb dhfrts**	
d106_8.F	5′ TAAAGAAGGCATAATTTTCA
DHFRp0.5R	5′ ACTTTATATTTTTCATTGAG
d106_8.RHEX	5′ CATTGAGATAAATAAGTGTTCA
-4.4kb dhfrts***	
DHFR4.4kb.3F	5' GTTGTCAATAATTTCTGCATC
DHFR4.4kb.R	5' CGATATATCTGATGGGTGA
DHFR4.4kb.RHEX	5'TACCATAGCAGTCTTTGCA

Pfmsp1 block2	
Universal primers	
Fmsp1uf (106-125[a])	5′ GAAGATGCAGTATTGACAGG
Fmsp1ur (586-607[a])	5′ CATTAATTTCTTCATATCCATC
Allelic specific primers	
k1ff (161-183[a])	5' ATGAAGAAGAAATTACTACAAAA
mad20ff (163-182[b])	5' GAAGGAACAAGTGGAACAGC
ro33ff (174-193[c])	5′ TACTCAAGTTGTTGCAAAGC

* locus also called 0.3kb primers from Roper et al, (2003) Lancet 361: 1174-1181

** primers d106_8.F and d106_8.RHEX from Nair S et al (2003) Mol Biol Evol 20: 1526-1536 DHFRp0.5R designed for this study

*** primers from Roper et al, (2003) Lancet 361: 1174-1181

[a,b,c] The *Pfmsp1 block2* positions are based on the reference strain Palo Alto-Uganda[a] (accession No M37213) for K1-type; MAD20[b] (accession No X05624) for MAD20-type and RO33Ghana[c] (accession No M35727) for RO33-type.
