# Supplementary material for: Rapid Dissemination of Plasmodium falciparum Drug Resistance Despite Strictly Controlled Antimalarial Use
Source: PLoS One. 2007 Jan 3;2(1):e139. doi: 10.1371/journal.pone.0000139 (PMC1764034; doi:10.1371/journal.pone.0000139)
Supplement: Table S2 — Pfcrt intron 4 Microsatellite haplotypes. (0.05 MB PDF) [file pone.0000139.s002.pdf]

Supplementary file S2 *Pfcrt* intron 4 Microsatellite haplotypes

Pfcrt haplotype code	*Pfcrt* intron 4 microsatellite sequence
1	(TAAA)2 (TA)14
2	(TAAA)2 (TA)15
3	(TAAA)2 (TA)16
4	(TAAA)2 (TA)23
5	(TAAA)2 (TA)24
6	(TAAA)3 (TA)7
7	(TAAA)3 (TA)9
8	(TAAA)3 (TA)10
9	(TAAA)3 (TA)11
10	(TAAA)3 (TA)12
11	(TAAA)3 (TA)13
12	(TAAA)3 (TA)14
13	(TAAA)3 (TA)15
14	(TAAA)3 (TA)16
15	(TAAA)3 (TA)17
16	(TAAA)3 (TA)18
17	(TAAA)3 (TA)19
18	(TAAA)3 (TA)20
19	(TAAA)3 (TA)21
20	(TAAA)3 (TA)22
21	(TAAA)3 (TA)24
22	(TAAA)4 (TA)7
23	(TAAA)4 (TA)8
24	(TAAA)4 (TA)10
25	(TAAA)4 (TA)11
26	(TAAA)4 (TA)14
27	(TAAA)4 (TA)15
28	(TAAA)4 (TA)20
29	(TAAA)4 (TA)21
30	(TAAA)4 (TA)22
31	(TAAA)4 (TA)23
