# Supplementary material for: Rapid Dissemination of Plasmodium falciparum Drug Resistance Despite Strictly Controlled Antimalarial Use
Source: PLoS One. 2007 Jan 3;2(1):e139. doi: 10.1371/journal.pone.0000139 (PMC1764034; doi:10.1371/journal.pone.0000139)
Supplement: Table S3 — Pfdhfr flanking microsatellite haplotypes. (0.06 MB PDF) [file pone.0000139.s003.pdf]

Supplementary File Table S3

Pfdhfr flanking microsatellite haplotypes

Individual haplotypes and corresponding codes are indicated. Note that for the –4.4kb locus was typed for a few isolates only and is undertemined except for haplotypes 9, 11, 16 ,29, 36 and 41.

		fragment length (bp)	
**Haplotype code**	**MS-4.4kb bp**	**MS-0.1Kb**	**MS+0.5kb**
**1**		87	91
**2**		87	95
**3**		87	97
**4**		87	101
**5**		87	103
**6**		87	109
**7**		89	97
**8**		95	91
**9**	169	95	95
**10**		95	97
**11**	178	95	103
**12**		95	111
**13**		97	101
**14**		97	103
**15**		99	91
**16**	180	99	97
**17**		99	99
**18**		99	103
**19**		99	107
**20**		99	109
**21**		99	111
**22**		103	85
**23**		103	93
**24**		103	95
**25**		103	101
**26**		105	109
**27**		107	89
**28**		107	93
**29**	175	107	105
**30**		109	79
**31**		109	93
**32**		109	105
**33**		111	85
**34**		111	93
**35**		111	95
**36**	175	111	97
**37**		111	103
**38**		111	109
**39**		114	103
**40**		115	97
**41**	163	118	97
**42**		118	107
**43**		122	103
**44**		122	109
controls	**MS-4.4kb bp**	**MS-0.1Kb**	**MS+0.5kb**
3D7 predicted	208	106	108
3D7 observed	not detected	103	105
FCC1	169	103	95
